# Changes in Lipids and Inflammatory Markers after Consuming Diets High in Red Meat or Dairy for Four Weeks

**DOI:** 10.3390/nu9080886

**Published:** 2017-08-17

**Authors:** Kirsty M. Turner, Jennifer B. Keogh, Peter J. Meikle, Peter M. Clifton

**Affiliations:** 1 Sansom Institute for Health Research, School of Pharmacy and Medical Sciences, University of South Australia, Adelaide, SA 5000, Australia; kirsty.turner@unisa.edu.au.com (K.M.T.); jennifer.keogh@unisa.edu.au (J.B.K.); 2 Baker IDI Heart and Diabetes Institute, Melbourne, VIC 3004, Australia; peter.meikle@bakeridi.edu.au

**Keywords:** red meat, dairy, insulin resistance, lipids, inflammation

## Abstract

There is a body of evidence linking inflammation, altered lipid metabolism, and insulin resistance. Our previous research found that insulin sensitivity decreased after a four-week diet high in dairy compared to a control diet and to one high in red meat. Our aim was to determine whether a relationship exists between changes in insulin sensitivity and inflammatory biomarkers, or with lipid species. Fasting Tumor Necrosis Factor alpha (TNF-α), Tumor Necrosis Factor Receptor II (sTNF-RII), C-reactive protein (CRP), and lipids were measured at the end of each diet. TNF-α and the ratio TNF-α/sTNF-RII were not different between diets and TNF-α, sTNF-RII, or the ratio TNF-α/sTNF-RII showed no association with homeostasis model assessment-estimated insulin resistance (HOMA-IR). A number of phosphatidylethanolamine (PE) and phosphatidylinositol (PI) species differed between dairy and red meat and dairy and control diets, as did many phosphatidylcholine (PC) species and cholesteryl ester (CE) 14:0, CE15:0, lysophosphatidylcholine (LPC) 14:0, and LPC15:0. None had a significant relationship (*p* = 0.001 or better) with log homeostasis model assessment-estimated insulin resistance (HOMA-IR), although LPC14:0 had the strongest relationship (*p* = 0.004) and may be the main mediator of the effect of dairy on insulin sensitivity. LPC14:0 and the whole LPC class were correlated with CRP. The correlations between dietary change and the minor plasma phospholipids PI32:1 and PE32:1 are novel and may reflect significant changes in membrane composition. Inflammatory markers were not altered by changes in protein source while the correlation of LPC with CRP confirms a relationship between changes in lipid profile and inflammation.

## 1. Introduction

Insulin resistance is defined as a reduced response of target tissues such as skeletal muscle, liver, and adipocytes to insulin [1]. Adipose tissue plays an important role in maintaining glucose homeostasis, however obesity results in an inflammatory state that contributes to insulin resistance [2]. With obesity rates on the rise around the world [3], it is important to understand how diet impacts the risk of developing insulin resistance as this condition increases the likelihood that other metabolic abnormalities will also be present, such as elevated plasma glucose, endothelial dysfunction, dyslipidemia, and increased inflammation [4].

With weight gain, adipocytes enlarge and secrete chemoattractants that recruit additional macrophages to the tissue [2]. Macrophages secrete pro-inflammatory cytokines such as tumor necrosis factor alpha (TNF-α), which can activate serine phosphorylation of insulin receptors, impairing insulin signalling and repressing genes involved in the storage of fatty acids and glucose [5], and in the differentiation of preadipocytes to adipocytes [6]. Interleukin-6 (IL-6), another pro-inflammatory cytokine released by adipocytes, in turn induces a rise in C-reactive protein (CRP) from the liver; elevated levels of these inflammatory markers have been shown to predict the onset of diabetes [7].

As dyslipidemia is associated with insulin resistance, high density lipoprotein (HDL) cholesterol and serum triglycerides are commonly abnormal in type 2 diabetes, but specific lipid species may also be useful in assessing risk [8]. Lipid profiling identified 135 lipids associated with type 2 diabetes (T2DM) and 134 with prediabetes in the Australian Diabetes, Obesity and Lifestyle Study (AusDiab), with most significant associations then validated in the San Antonio Family Heart Study (SAFHS) [9].

While a high consumption of red meat has been associated with an increased risk for T2DM [10], high dairy intake has been associated with lower risk factors, including body weight [11], insulin resistance [12], and inflammation [13], as well as a lower risk of incident T2DM [14]. Results from interventions are mixed however [15,16,17]. Our research evaluating the effect of three weight-stable diets on glucose metabolism [18] found that insulin sensitivity decreased by 16% after a four-week diet high in dairy in comparison to a control diet and to one high in red meat, particularly in women. Our aim therefore was to determine if there is any relationship between the changes in insulin sensitivity observed in this study and the inflammatory biomarkers CRP, TNF-α, and soluble Tumor Necrosis Factor Receptor II (sTNF-RII) and whether there were any correlations with specific lipid species.

## 2. Materials and Methods

### 2.1. Participants

Participants were recruited by public advertisement and screened for eligibility. Details of all inclusion and exclusion criteria, and the effects on insulin sensitivity (the primary outcome measure for this trial) have been reported previously [18]. Participants had a 75 g oral glucose tolerance test to determine glycemic status and exclude those with frank diabetes. The University of South Australia Human Research Ethics committee approved the study and all participants provided written informed consent prior to participating (Ethic Code No. 31372). The trial was registered with the Australian New Zealand Clinical Trials Registry (ACTRN12613000441718). An amount of AUD$150 was offered to the participants at completion of the study.

### 2.2. Dietary Intervention

During the high red meat diet, participants were instructed to consume at least 200 g of red meat per day, six days per week and to consume minimal (less than one serving) of dairy per day. During the high dairy diet, participants were instructed to consume 4–6 servings of primarily low-fat dairy (from milk, yogurt, custard and cheese) per day with chicken and fish as additional sources of protein but no red meat. Serving sizes were defined using the guidelines of the Australian National Health and Medical Research Council. The low dairy, no red meat control diet contained at least 200 g of fish or chicken each day, with less than one serving/day of dairy. Participants attended the clinic on three occasions during each diet in order to monitor weight and ensure dietary compliance. Participants were asked not to consume processed meat for the duration of the study. Diet order was randomized with all participants completing each four-week diet with a two-week washout period in between. Verbal and written instructions, including explanations of serving sizes, were provided for each diet, along with digital kitchen scales (Homemaker Slimline Electronic Scale, Shenzhen Hostweigh Electronic Technology Co., Ltd., Shenzhen, China).

### 2.3. Dietary Measurements

A daily checklist was completed during each dietary period in order to obtain the amount of red meat, dairy or alternate protein sources consumed each day. A three-day weighed food record was also completed within each two-week period. All food and beverages consumed over these three-day weighed periods were recorded and entered into Food Works Professional Edition 7.0 (Xyris, Brisbane, Australia) for dietary analysis.

### 2.4. Clinical Measurements

Height was measured on a wall mounted stadiometer (Seca, Hamburg, Germany) at the baseline visit. Body weight was measured at each visit using electronic digital scales (Tanita Corporation, Tokyo, Japan) in light clothing and without shoes. Body mass index (BMI) was calculated by weight (kg) divided by height (m). Body composition was assessed at baseline by whole-body dual energy X-ray absorptiometry (DEXA) (Lunar Prodigy, Lunar Radiation Corp., Madison, WI, USA). After an overnight fast, participants came to the Sansom Institute for Health Research Clinical Trial facility at the University of South Australia for an 75g oral glucose tolerance test (OGTT). These were performed at the end of each diet with blood samples taken every 30 min for a total of five time points. Blood for serum was collected in tubes with no additives and allowed to clot at room temperature for 30 min. Blood for plasma was collected in Sodium Fluoride ethylenediaminetetra-acetic acid (EDTA) tubes and stored on ice until processed. Blood samples were separated by centrifuge at 4000 RPM at 4 °C for 10 min (Universal 32R, Hettich Zentrifugen, Hettich, Tuttlingen, Germany).

Serum hsCRP, triglycerides, total cholesterol, and HDL cholesterol were measured using an automated spectrophotometric analyzer (Konelab 20XTi, Thermo Electron, Waltham, MA, USA) and serum tumor necrosis factor alpha (TNF-α) and soluble tumor necrosis factor alpha receptor 2 (sTNF-RII) were measured by commercial ELISA kits (Invitrogen Corporation, Carlsbad, CA, USA Kit # KHC3013 for UltraSensitive TNF-α, Kit # KAC1771 for sTNF-RII). Lipid analysis was performed as outlined in Weir et al. [19] by LC ESI-MS/MS using an Agilent 1200 liquid chromatography system and Applied Biosystems API 4000 Q/TRAP mass spectrometer with a turbo-ion spray source (350 °C) and Analyst 1.5 and Multiquant data systems. Liquid chromatography was performed on a Zorbax C18, 1.8 m, 50 × 2.1 mm column (Agilent Technologies, Santa Clara, CA, USA). Total lipid classes were calculated the sum of all the individual measured species in that class.

### 2.5. Analysis

Statistical analysis was performed using SPSS V22 (IBM, Chicago, IL, USA). Data were examined for normality before analysis; variables not normally distributed (HOMA-IR and lipids) were log transformed prior to testing. The power calculation was based on our primary outcome of insulin sensitivity with a sample size of 45 providing 90% power (alpha 0.05) to see a 20% change in insulin sensitivity as assessed by both HOMA-IR and Matsuda’s index. As neither the Matsuda nor Stumvoll indices were different between the diets, only HOMA-IR was used as an index of insulin sensitivity. Although there were sex differences in relation to insulin and HOMA-IR: red meat compared with dairy for insulin: diet, *p* = 0.02 (higher for dairy); diet by sex, *p* = 0.02 (higher in dairy for women) and red meat compared with dairy for HOMA_IR diet (higher for dairy), *p* = 0.04; diet by sex, *p* = 0.03 (higher in dairy for women) adjustment for sex had no effect on our other analyses.

As the primary endpoint of insulin sensitivity differed only between dairy and red meat and dairy and control diet the potential mediators were analysed by paired t-tests between dairy (DD) and control (CD) and dairy (DD) and red meat (RM) diets. Red meat was not compared to the control. Lipid species from the 319 species measured by LC-MS were selected for analysis on the following basis: (1) species containing fatty acids known to be found in dairy fat 15:0, 17:0, and 16:1 and that have been previously related to dairy intake [20]; (2) lipid species associated with insulin sensitivity in other cross-sectional studies: Lysophosphatidylcholine (LPC) 15:0 and 17:0 and lysoplatelet activating factor (LPC(O)) 20:0 and 22:1 and c16:0 ceramide [21]; (3) lipid species that were found to be significantly different in both the dairy/control diet and dairy/red meat diet contrasts, with at least one of these contrasts having a p value of 0.001; and (4) lipid species found to be different between normal individuals and those with prediabetes in the AusDiab study [9]. From the AusDiab results, we selected species that had a risk ratio of >1.5 or <0.66 not previously identified using criteria 1–3. We thus selected 42 lipid species for further analysis. Given the number of comparisons, we designated *p* ≤ 0.001 as statistically significant for relationships with log HOMA. Relations with log HOMA were assessed by a one way ANOVA, with subject ID as a fixed factor and log lipid species as a covariate to perform intra-individual correlations. Relationships between TNF alpha and CRP and HOMA-IR were performed in the same way. For relationships with BMI, glucose tolerance status variables were averaged across the three diets and a Pearson correlation performed. Relationships between lipids and inflammatory markers were regarded as exploratory and were not fully adjusted for multiple comparisons and *p <* 0.01 was regarded as significant.

## 3. Results

Forty-seven people completed the study. The baseline characteristics of each group are shown in Table 1. Energy intake was higher with the dairy diet (Table 2) than with both red meat and control diets (*P*-both comparisons < 0.001) and total and saturated fat intakes were also higher during the dairy diet than during either the red meat or control diet (*P*-both comparisons < 0.01). Carbohydrate intake was similarly higher with the dairy diet than with either the red meat or control diet (*P*-both comparisons < 0.001). Adjustment for carbohydrate did not abolish the diet effect on HOMA-IR. The dairy diet resulted in a small weight gain from the start of the diet (0.1 ± 1.2 kg), whereas red meat and control diets each resulted in a loss of 0.4 ± 1.1 kg. Men had a greater decrease in weight during red meat and control diets than women did (data not shown; *P*-change < 0.05), however, the weight change between diets did not have a significant effect on any of the sensitivity indexes for the group as a whole or when analyzed by group or sex. Similarly, energy intake and total and saturated fat intakes were unrelated to changes in insulin sensitivity and inflammatory markers.

TNF-α, sTNF-RII, and the ratio TNF-α/sTNF-RII were not different between diets (Table 3) and TNF-α, sTNF-RII, or the ratio TNF-α/sTNF-RII showed no association with HOMA-IR. CRP was not different between diets but within subjects across all diets CRP was weakly positively associated with TNF-α (*r* = 0.25, *p* = 0.013). CRP was strongly correlated with BMI (*r* = 0.502, *p <* 0.001) but not with glucose tolerance. TNF-α was not associated with BMI or with glucose tolerance.

Total, HDL and LDL cholesterol and triglycerides were not different between diets, nor were they related to HOMA-IR. There was no effect of age, % fat mass, or glucose tolerance group when added as covariates. Results from individual lipid species and changes between diets are shown in Table 4. In the cholesterol ester (CE) group CE 15:0 and CE 14:0 differed between both dairy and red meat and dairy and control diets, while CE 16:1 and CE 16:2 differed only between dairy and control and this cannot explain the overall findings. CE 17:0 was not different between diets and none of the CE species were related to insulin sensitivity as assessed by log HOMA. A number of phosphatidylethanolamine (PE) and phosphatidylinositol (PI) species differed between both the dairy and red meat and the dairy and control diets, as did many of the phosphatidylcholine (PC) species and LPC 14:0 and LPC 15:0. None had a significant relationship (a priori defined as *p* = 0.001) with log HOMA, although LPC 14:0 had the strongest relationship (*r* = 0.3, *p* = 0.004). PE 32:1 and PI 32:1 were increased in the dairy diet compared with both the red meat and control diets and were good predictors of prediabetes in the AusDiab study. The relationship between log HOMA-IR and lipids was in the same direction as seen in the AusDiab prediabetes predictor lipids. Glycemic status (NGT or IFG/IGT) did not influence the changes in lipids between diets nor the relationship between log HOMA-IR and lipids. The alkylphosphatidylcholine (PC(O)) and alkenylphosphatidylcholine (PC(P)) classes differed between both dairy and red meat and dairy and control diets, but none of the overall classes were related to insulin resistance. Table 5 shows associations between inflammatory markers and lipid species. LPC 14:0 and the whole LPC class were significantly associated with CRP.

## 4. Discussion

In this study we compared the effects of three weight stable diets: a high dairy diet, a high meat diet and a control diet containing no dairy or red meat consumed for four weeks each in 47 people about half of whom had impaired glucose tolerance of impaired fasting glucose. We found that the dairy diet was associated with higher fasting insulin and higher HOMA-IR than the other two diets. The purpose of this paper was to explore possible mechanisms for this finding focused on inflammatory markers and circulating lipid species.

CRP, TNF-α, and the ratio TNF-α/sTNF-RII were not different between any of the diets and there was no association with insulin sensitivity. A few studies have assessed the effect of red meat on inflammatory markers. Red meat was directly correlated with CRP levels in a cross-sectional study in Tehran, however the FFQ used to estimate intake did not separate out processed red meat in the analysis [22]. Processed meat was positively correlated with CRP levels in a sub-cohort of the Rotterdam study but lean red meat and chicken were not associated [23]. Partially replacing carbohydrates with 200 g of lean red meat in an eight-week parallel study comparing two isoenergetic diets found no increase in inflammation or oxidative stress [24]. CRP levels were associated with a high consumption of red meat in a cross-sectional analysis of a European Prospective Study into Cancer (EPIC) sub-cohort, but after adjusting for body mass index and waist circumference this association was not significant, indicating that obesity was responsible for the elevated levels [25]. Thus, the majority of studies confirm our finding of no association between lean red meat intake and inflammatory markers.

Dairy consumption has been reported to reduce inflammatory stress but the results from intervention studies have been mixed. TNF-α and CRP levels were similar after each of the four-week diets in the present study, whereas consuming dairy-based smoothies led to reductions in TNF-α, IL-6, and MCP-1 compared with soy-based smoothies in a crossover trial of 40 overweight and obese adults [26]. Similarly, significant reductions in TNF-α, MCP-1, IL-6 and CRP were also observed in overweight and obese individuals after following an adequate dairy (>3.5 servings/day) compared to those following a low dairy (<0.5 servings/day) weight maintenance diet, but a significant decrease in fat mass in those consuming more dairy is likely to have been responsible for these results [13]. Participants with metabolic syndrome who added three servings of dairy per day for six weeks found a reduction in TNF-α and MCP-1 when compared to a diet that contained energy matched control foods, but the decrease was only observed in women [27]. Women also lost weight during the dairy period which may have influenced the results. No change in inflammatory markers was observed for men, although a reduction in fasting glucose was observed (*p* = 0.048), but HOMA-assessed insulin sensitivity did not change for either men or women [28]. In contrast, a study comparing low fat dairy (milk and yogurt) with a carbohydrate control (fruit juice and biscuits) found no difference in IL-6 or MCP-1 between groups and, while TNF-α trended lower in the dairy period, the difference was not significant [29]. Wennersberg et al. similarly showed no effect of a six-month dairy intervention on IL-6, CRP, or TNF-α [30]. Overall, we can conclude that dairy does not reduce inflammation and inflammatory markers are not associated with insulin resistance in dairy interventions.

Concentrations of many of the ether-linked phospholipids alkylphosphatidylcholine (PC(0)) and alkenylphosphatidylcholine (PC(P)) were significantly lower after the dairy diet than either the red meat or control diet and there was a negative association with log HOMA for some species, linking increased insulin resistance after the dairy diet to these species, although this did not meet the pre-planned 0.001 level of significance. Low levels of these species were also found in the AusDiab pre-diabetes population [9]. These are relatively minor plasma species however.

Phosphatidylethanolamines (PE) and phosphatidylinositols are also minor species in plasma, but they are important structural lipids in membranes [31]. Polyphosphoinositides (PI), phosphorylated forms of phosphatidylinositol, play important roles in cell signaling and in the regulation of membrane traffic and transport functions [32] and also serve as precursors for second messengers [33]. The most common membrane form of phosphatidylinositol is one containing stearic acid in the sn1 position and arachidonic acid in the sn2 position [34,35] and is a major source of arachidonic acid for prostaglandins and leukotrienes, important mediators of inflammatory response [36]. Both PE 32:1 and PI 32:1 were significantly higher after the dairy diet than after both red meat and control diets and trended toward a positive correlation with log HOMA but did not reach our pre-determined level of significance. Both of these species were higher in the AusDiab prediabetes population compared with the control group [9], which is consistent with our findings. This may be reflective of changes in membrane composition but whether as a result of insulin resistance or as a contributor to it is not known.

Cholesterol esters (CE) are the most abundant class of plasma lipids [37] and in the present study were found to be higher after the dairy diet, although not significantly. The higher fasting insulin levels on the dairy diet may have stimulated de novo synthesis of fatty acids, as reflected by the higher CE 16:1 levels, a biomarker of lipogenesis [38]. CE 16:1 and CE 16:2 were not associated with log HOMA in this study, although they were associated with pre-diabetes and type 2 diabetes in the AusDiab study [9].

Lysophosphatidylcholine (LPC) 15:0 and 17:0 have previously been associated with full fat dairy intake and inversely correlated with insulin resistance [21]. While LPC 15:0 was higher after the dairy diet in the present study, LPC 17:0 was similar after both red meat and dairy, as expected since the fatty acid composition of dairy and beef fat is similar except for a higher myristic acid (C14:0) in dairy [39]. Neither LPC 15:0 and LPC 17:0 species was associated with insulin resistance but LPC 14:0 and the overall LPC class were associated with CRP at our pre-determined *p <* 0.01 level. LPC is formed during LDL oxidation and while LPC constitutes 1–5% of total PC content of non-oxidized LDL, during oxidation as much as 40–50% of PC may be converted to LPC [40]. Higher LPC levels have been shown to release inflammatory cytokines [41] and recruit monocytes and pro-inflammatory cytokines to atherosclerotic lesions [42]. LPC levels have been shown to be elevated in obesity [43] and were 2.8 times higher for those with diabetes in comparison to non-diabetic controls [44]. LPC 14:0 was significantly higher after the dairy diet compared with the red meat diet and the control diet and the association with log HOMA trended toward significance (*p* = 0.004), adding further to the body of evidence linking inflammation, altered lipid metabolism, and insulin resistance [45,46]. LPC14:0 may be the main mediator of the effect of dairy on insulin sensitivity seen in this study.

While it appears that adherence to the protocol was met, a limitation of the study is that self-reporting of food intake is known to be problematic, with participants potentially reporting according to expected demand instead of actual intake [47]. Red meat may also be very lean compared with usual population intakes. Also, the study was powered for our primary outcome of insulin sensitivity and it is possible we may have been underpowered to detect changes in inflammatory markers between diets. A final limitation is that in endeavoring to ensure our type 1 errors are reduced we have introduced several type 2 errors and that changes in LPC 14:0 may reproducible in future studies.

## 5. Conclusions

In conclusion, we have observed that inflammatory markers were not altered by changes in protein source. We confirmed earlier findings of correlations between LPC and inflammatory markers, and while the association with log HOMA was modest, the correlations between dietary change and the phospholipid species PI 32:1 and PE 32:1 are novel findings that may reflect changes in membrane composition that occur in insulin resistance.

## Figures and Tables

**Table 1:** Baseline characteristics of participants ^1^.

	NGT	IFG/IGT
Sex M/F	12/15	6/14
Age *	44.3 ± 12.9	52.5 ± 12.0
BMI (kg/m^2^)	30.7 ± 4.1	31.6 ± 6.3
Baseline SBP (mmHg)	124.9 ± 16.8	128.9 ± 12.9
Baseline DBP (mmHg)	81.7 ± 10.3	83.6 ± 7.9
^1^ Total Fat Mass (%)	39.8 ± 9.3	38.3 ± 9.2
^1^ Total Lean Mass (%)	60.6 ± 9.4	61.7 ± 9.2
^1^ Total Fat Mass (kg)	35.3 ± 10.5	29.8 ± 7.6
^1^ Total Lean Mass (kg)	50.6 ± 10.0	45.7 ± 9.9

^1^ *n* = 45. * Significantly different between groups (*p <* 0.05). All values are mean ± SD. SBP systolic blood pressure, DBP diastolic blood pressure

**Table 2:** Macronutrient composition of the three diets.

	Red Meat	Dairy	Control
Energy, kJ	8205 ± 1840 ^a^	9332 ± 1525 ^b^	7811 ± 1946 ^a^
Protein, g	118 ± 23 ^a^	118 ± 24 ^a^	103 ± 20 ^b^
Total fat, g	74 ± 21 ^a^	85 ± 20 ^b^	69 ± 19 ^c^
Saturated fat, g	25 ± 9 ^a^	39 ± 11 ^b^	21 ± 9 ^c^
Carbohydrate, g	182 ± 55 ^a^	231 ± 56 ^b^	186 ± 70 ^a^
Dietary fiber, g	26 ± 9 ^a^	23 ± 8 ^b^	25 ± 10 ^a,b^
Calcium, mg	485 ± 168 ^a^	1763 ± 303 ^b^	533 ± 225 ^c^
Kilojoules from protein, %	25 ± 4 ^a^	22 ± 4 ^b^	23 ± 5 ^c^
Kilojoules from fat, %	34 ± 6	34 ± 6	33 ± 7
Kilojoules from saturated fat, %	12 ± 3 ^a^	15 ± 4 ^b^	10 ± 3 ^c^
Kilojoules from carbohydrate, %	36 ± 6 ^a^	40 ± 6 ^b^	38 ± 8 ^c^
Kilojoules from fiber, %	3 ± 0.8 ^a^	2 ± 0.6 ^b^	3 ± 0.7 ^a^
Fat as saturated, %	37 ± 6 ^a^	49 ± 7 ^b^	34 ± 7 ^c^

All values are means ± SDs. Values in a row that do not share a common superscript letter are significantly different, *p* < 0.05 (3-diet repeated-measures ANOVA).

**Table 3:** Effect of diet on inflammatory markers.

	Red Meat	Dairy	Control
hsCRP mg/L	5.25 ± 7.95	5.03 ± 8.10	4.04 ± 6.88
TNFα pg/mL	1.40 ± 0.93	1.45 ± 1.01	1.45 ± 1.02
sTNFRII pg/mL	4.94 ± 2.76	4.68 ± 2.63	4.48 ± 2.49
Ratio TNFα/sTNFRII	0.34 ± 0.33	0.37 ± 0.32	0.38 ± 0.31

Abbreviations: hsCRP: high sensitivity C-reactive protein; TNF-α: tumor necrosis factor alpha; sTNF-RII: Tumor Necrosis Factor Receptor II. All values are mean ± SD. *n* = 47 paired t tests between dairy and red meat diet and between dairy and control diet all not significant (*p <* 0.01).

**Table 4:** Lipid comparisons between diets and correlations with log HOMA.

	Red Meat	Dairy	Control	T-Test	T-Test	Correlation	Aus Diab Assn
	nmol/mL	nmol/mL	nmol/mL	RM/DD	DD/CD	Wi th Log HOMA	with PreDiabetes ^1^
				(*p* value)	(*p* value)	(*p* value)	(0R)
**CE 14:0**	14 (9–20)	17 (13–24)	13 (8–18)	0.009	**<0.001**	0.73	2.2 (1.4–3.3)
**CE 15:0**	21 (17–27)	24 (22–33)	18 (13–24)	0.003	**<0.001**	0.55	1.4 (0.9–2.1)
**CE 16:1**	86 (68–134)	98 (72–135)	85 (60–111)	0.22	0.004	0.56	2.1 (1.4–3.1)
**CE 16:2**	3.8 (2.0–5.0)	4.3 (3.1–5.5)	3.6 (2.7–4.7)	0.15	0.002	0.53	2.3 (1.5–3.5)
**CE 17:0**	179 (163–205)	188 (172–205)	177 (162–207)	0.89	0.032	0.65	0.93 (0.6–1.4)
**LPC 14:0**	1.2 (0.9–1.5)	1.4 (1.2–1.6)	1.1 (0.9–1.3)	0.004	**<0.001**	0.004	1.7 (1.2–2.4)
**LPC 15:0**	0.7 (0.6–0.8)	0.8 (0.6–0.9)	0.6 (0.5–0.7)	0.03	**<0.001**	0.055	1.1 (0.7–1.6)
**LPC 17.0**	1.4 (1.1–1.8)	1.4 (1.2–1.6)	1.2 (0.9–1.4)	0.092	**<0.001**	0.46	
**PC 16:0/20:4**	114 (102–140)	112 (92–131)	113 (96–138)	0.003	0.002	0.62	
**PC 28:0**	0.3 (0.1–0.4)	0.4 (0.2–0.5)	0.2 (0.1–0.3)	0.002	**<0.001**	0.005	
**PC 30:0**	3.4 (2.4–4.1)	4.3 (3.1–5.7)	3.1 (2.2–3.9)	0.003	**<0.001**	0.010	
**PC 38:4**	82 (67–93)	75 (62–91)	79 (66–95)	**<0.001**	**<0.001**	0.30	
**PC(O-34:2)**	4.8 (3.8–5.7)	3.8 (2.8–4.2)	4.1 (3.6–4.6)	**<0.001**	**<0.001**	0.73	0.60 (0.38–0.96)
**PC(O-18:1/18:1)**	0.8 (0.6–0.9)	0.6 (0.5–0.8)	0.7 (0.5–0.9)	**<0.001**	0.012	0.080	
**PC(O-18:1/18:2)**	3.2 (2.9–3.7)	2.8 (2.3–3.2)	3.2 (2.8–3.5)	**<0.001**	**<0.001**	0.47	
**PC(O-36:4)**	13 (11–15)	10 (8–12)	11 (10–13)	**<0.001**	**<0.001**	0.22	0.63 (0.42–0.92)
**PC(O-38:5)**	12 (11–15)	11 (9–12)	13 (11–14)	**<0.001**	**<0.001**	0.20	0.58 (0.37–0.90)
**PC(O-40:5)**	1.7 (1.4–2.0)	1.4 (1.2–1.7)	1.6 (1.3–1.8)	**<0.001**	0.004	0.022	
**PC(O-40:6)**	1.3 (1.1–1.7)	1.1 (0.9–1.4)	1.2 (1.0–1.5)	**<0.001**	**<0.001**	0.093	
**PC(O-40:7)**	2.7 (2.1–3.3)	2.3 (1.8–2.99)	2.9 (2.3–3.7)	0.001	**<0.001**	0.50	
**PC(P-34:1)**	2.4 (1.9–2.9)	2.0 (1.7–2.4)	2.1 (1.8–2.6)	**<0.001**	0.006	0.076	0.57 (0.36–0.91)
**PC(P-34:2)**	7.9 (6.6–9.6)	6.0 (5.2–6.8)	6.4 (5.5–7.4)	**<0.001**	0.002	0.29	
**PC(P-36:2)**	1.5 (1.1–2.1)	0.8 (0.6–1.2)	0.9 (0.8–1.2)	**<0.001**	**<0.001**	0.29	0.53 (0.34–0.84)
**PC(P-36:4)**	9.8 (8.3–11.0)	6.6 (5.6–7.4)	7.8 (6.3–9.1)	**<0.001**	**<0.001**	0.082	
**PC(P-38:4)**	2.5 (2.0–3.3)	1.4 (1.1–1.7)	1.6 (1.3–2.1)	**<0.001**	**<0.001**	0.10	
**PC(P-38:5)**	5.7 (4.7–6.9)	4.6 (4.0–5.5)	5.2 (4.7–6.5)	**<0.001**	**<0.001**	0.26	
**PC(P-38:6)**	1.3 (1.1–1.8)	0.9 (0.7–1.3)	1.2 (0.9–1.6)	**<0.001**	**<0.001**	0.21	
**PC(P-40:6)**	0.6 (0.5–0.9)	0.4 (0.3–0.6)	0.5 (0.4–0.6)	**<0.001**	**<0.001**	0.15	
**PE 32:1**	0.04 (0.02–0.08)	0.07 (0.04–0.12)	0.04 (0.03–0.07)	**<0.001**	**<0.001**	0.027	1.5 (1.1–2.1)
**PE(O-18:1/20:3)**	0.5 (0.4–0.6)	0.3 (0.2–0.4)	0.4 (0.3–0.6)	**<0.001**	0.001	0.20	
**PE(O-18:2/18:2)**	1.0 (0.8–1.3)	0.9 (0.6–1.2)	1.1 (0.9–1.3)	0.002	**<0.001**	0.59	
**PE(P-16:0/20:4)**	6.6 (5.2–7.7)	5.4 (4.5–7.1)	6.5 (5.5–8.0)	0.005	**<0.001**	0.76	
**PE(P-18:0/20:4)**	12 (9–14)	7.4 (5.6–9.0)	8.6 (7.5–10.8)	**<0.001**	**<0.001**	0.37	
**PE(P-18:0/22:6)**	2.8 (2.2–4.0)	2.2 (1.0–3.1)	2.7 (2.2–3.4)	**<0.001**	**<0.001**	0.26	
**PE(P-18:1/20:4)**	6.3 (5.2–7.4)	4.8 (3.7–6.1)	6.3 (5.3–7.3)	**<0.001**	**<0.001**	0.57	
**PE(P-20:1/20:4)**	0.2 (0.1–0.2)	0.3 (0.2–0.3)	0.3 (0.2–0.4)	**<0.001**	**<0.001**	0.45	
**PE(P-20:1/22:6)**	0.06 (0.04–0.09)	0.08 (0.06–0.12)	0.1 (0.08–0.16)	**<0.001**	**<0.001**	0.77	
**PI 32:0**	0.3 (0.2–0.5)	0.4 (0.3–0.8)	0.3 (0.2–0.6)	0.02	**<0.001**	0.009	1.5 (1.1–2.1)
**PI 32:1**	0.5 (0.3–1.0)	0.8 (0.4–1.5)	0.6 (0.3–1.0)	0.002	**<0.001**	0.070	1.9 (1.3–2.7)
**PI 20:4/0:0**	0.4 (0.3–0.5)	0.4 (0.3–0.5)	0.4 (0.4–0.5)	**<0.001**	**<0.001**	0.23	
**SM 31:1**	0.3 (0.2–0.4)	0.4 (0.3–0.5)	0.3 (0.2–0.4)	0.002	**<0.001**	0.20	
**SM 32:1**	13 (10–15)	14 (12–18)	12 (10–16)	0.003	**<0.001**	0.34	
**PCO Class**	59 (528–68)	48 (44–57)	54 (48–58)	**<0.001**	0.005	0.52	
**PCP Class**	35 (30–44)	26 (23–29)	29 (26–32)	**<0.001**	**<0.001**	0.26	
**PEP Class**	49 (44–63)	41 (34–50)	46 (41–52)	**<0.001**	0.006	0.71	

^1^ Two separate paired t tests were completed to compare lipid values on the dairy diet (DD) and the red meat (RM) and control diet (CD) after log transformation. Values shown are medians and interquartile ranges. In the AusDiab study the odds ratio (OR) was derived from logistic regression of the prediabetes group (*n* = 64) versus the normal glucose tolerance group (*n* = 168) for an interquartile range increase in lipid predictor. The regression was adjusted for age, sex, waist circumference, and SBP. Relations with log HOMA were assessed by one way ANOVA with subject ID as a fixed factor and log lipid species as a covariate to perform intra-individual correlations.

**Table 5:** Correlations between lipid species and inflammatory markers TNF-α, sTNFRII and hsCRP.

	hsCRP	TNFα	sTNFRII
LPC 14:0	0.008	0.033	0.028
LPC 15:0	0.034	0.25	0.23
LPC 17.0	0.040	0.21	0.72
PC 28:0	0.078	0.027	0.053
PC 30:0	0.045	0.066	0.10
PE(P 20:1/20:4)	0.021	0.87	0.027
PE(P 20:1/22:6)	0.10	0.68	0.048
LPC	0.001	0.28	0.062
PC	0.030	0.44	0.42
PE(P)	0.029	0.44	0.38
TG	0.044	0.74	0.61
PG	0.047	0.88	0.35
COH	0.050	0.67	0.85

Abbreviations: hsCRP: high sensitivity C-reactive protein; TNFα: tumor necrosis factor alpha; sTNF-RII: Tumor Necrosis Factor Receptor II. Relations were assessed by one way ANOVA with inflammatory markers as the dependent variable, subject ID as a fixed factor and log lipid species as a covariate.
